# Librarians against scientists: Oncotarget’s lesson

**DOI:** 10.18632/oncotarget.24272

**Published:** 2018-01-19

**Authors:** Mikhail V. Blagosklonny

Before the Internet, libraries played a crucial role in the dissemination of information and ruled the publishing industry: publishers begged librarians to subscribe to their journals. Publishers maintained official and personal contacts to ensure that librarians subscribed to their journals. Librarians were the bosses, with real power. But the internet and other technological advances changed the game. Librarians lost power and funding. Essentially, traditional libraries were becoming obsolete. Scientists could read journals, especially open access journals, at their workplace without going to a library. Open access papers cost many times less for society. Libraries are trying to adapt to this new reality by assuming new functions. Unfortunately, some older generations of librarians have worked to turn back technological progress and are fighting to regain their power. **Formally, they announced a war against open access journals, but, in reality, they announced a war against publishers who do not retain subscription journals.**

For example, to the best of my knowledge, Jeffrey Beall, a librarian, did not put on his blacklist prominent publishers of open access journals who also publish subscription journals. He does not care about open access journals, per se. It is simple: if publishers retain subscription journals, if they remain on their knees asking librarians to subscribe to their journal at enormous cost to society (in addition to fee charged the authors for publication), they are not put on the blacklist. In that situation, a librarian, such as Jeffrey Beall, is the boss. Moreover, a question that has puzzled people for a decade is, why are all of a publisher’s journals blacklisted, regardless of substantial differences among those journals? Why the entire publisher? Never half the journals of the publisher: either all the journals or no journal is blacklisted (there may be exceptions I am not aware of).

For example, all the publications of Impact Journals LLC were put on Beall’s blacklist without explanation in 2015. What is the common thread between Oncotarget and Oncoscience? Oncotarget is a large and prominent journal, whereas Oncoscience is a small journal that is free for readers and authors alike. It is a kind of charity journal, so to speak, and cannot possibly be predatory because it is intentionally losing money for the benefit of humanity. **The common thread is this: these journals are both published by the same publisher, who does not publish subscription journals, and therefore does not beg on its knees for libraries to subscribe.** Beall is losing power and therefore chose Oncotarget as a principle target for an attack. Oncotarget was extremely successful, easily competing with other cancer journals, including mega-journals (see Oncotarget Home page). This was likely seen as a very bad example to others. It shows that a new type of journal can be extremely successful, and it was therefore decided that it should be destroyed. Over a period of two years, Beall and anonymous bloggers have harassed Oncotarget and its Editor–in-Chief personally. We were harassed on many levels, daily, including defamation in a Wikipedia controlled by Beall’s co-fighters.

Despite this harassment, Oncotarget flourished, showing how resilient an honest journal can be. The main attack occurred in August 2017: several librarians at the National Library of Medicine (NLM), including J. Backus, decided to selectively re-evaluate Oncotarget without warning. They requested no information from us before deleting Oncotarget from MEDLINE. It is an unprecedented case for such a prominent journal. Remarkably, **according to the statement from the MEDLINE reviewers on 6/22/2017, “This journal [Oncotarget] continues to play a major role in the publication of important basic science research papers. Editorial practices are consistently high. Ethical guidelines are consistently followed. This is an important research journal for the field.”** The journal was described as “excellent.” It is noteworthy that MEDLINE’s decision to delete Oncotarget was based in part on incorrect information. For example, they stated that we do not publish clinical papers, which of course we do. The MEDLINE decision was invalid for many reasons, including technicalities.

Ultimately, MEDLINE deleted Oncotarget from PubMed Central and PubMed even without re-evaluation. They merely incorrectly informed us that we had never been accepted to PubMed Central, although we had been and we posted all our issues there. Numerous papers in Oncotarget were cynically deleted from PubMed based solely on the date of acceptance, thereby hurting science and the scientists, wasting taxpayer money (as these papers re-appeared on PubMed later), and preventing scientists from disseminating their research. Later Backus and Beall gave a misleading interview that appeared online. Pro-Beall bloggers filled the Internet with misinformation about the event. Only the actions of the leadership of the NLM stopped this injustice and restored our PubMed and PubMed Central status, but they did not have enough power to immediately reverse the MEDLINE decision. We remain hopeful that the decision will be reversed.

Despite these attacks, Oncotarget has continued to serve as a well-recognized and respected international journal, and has continued to flourish. As an example, **Web of Science (Clarivate Analytics) recently chose Oncotarget as the best journal in their rubric “Rising Star” out of 10 thousand journals, based on their serious analysis of data.** Unexpectedly, however, in January 2018 we started to hear rumors from China that Clarivate Analytics had stopped indexing Oncotarget after the November 10, 2017 issue. Once again, this action was taken without informing Oncotarget and without asking for any information from us. We sent several emails in January and did not get a response. Finally, we were able to reach high level executives, who agreed to index Oncotarget up to January 2018, but would stop indexing for 2018. When asked for their concerns, they failed to respond to our rebuttal, despite their promises to do so. Clarivate benefits from libraries’ subscriptions, so the entire system works together. The action of Clarivate was cynical, and once again the authors are suffering. We are hopeful that we can work with Clarivate so that the indexing of Oncotarget can be expeditiously resumed.

For thousands of years libraries served science and scientists. Indexes were created to disseminate information, not to suppress it. For the first time in history, in response to rapid technological progress, librarians are suppressing science and refusing to serve science. Of course, this not true of all librarians. The new generation of librarians resist Beall’ s ideas, including Beall’s supervisor, whose recent article should be read by everyone.

We, the scientists, should change the situation and change the policies of Indexes such as Clarivate’s Web of Science and MEDLINE, and make indexes work for us. Otherwise, why should these organizations exist?

## 

### PS:

The link to this Editorial was sent to Retraction Watch as a referral to their inquiry about de-listing Oncotarget from Web of Science.

