# Addendum: Librarians against scientists: Oncotarget’s lesson

**DOI:** 10.18632/oncotarget.24692

**Published:** 2018-03-13

**Authors:** Mikhail V. Blagosklonny

**This article has an addendum:** The below information has been added to this article. https://crln.acrl.org/index.php/crlnews/article/view/16837/18434

Original article: Oncotarget. 2018; 9:5515-5516. https://doi.org/10.18632/oncotarget.24272

